# Defining the optimal Ki67 cutoff values for survival prediction in neoadjuvant chemotherapy-treated patients with breast cancer

**DOI:** 10.3389/fsurg.2025.1697963

**Published:** 2025-11-24

**Authors:** Chun Jiang, Tong Zhu, Yunfei Zong, Ruibin Liu, Jiang Du, Junze Dai, Yuxuan Song, Dingye Zhang, Xin Wang, Zhaohu Shi, Yinping Jiang, Jiawen Bu, Baifang Ding, Xudong Zhu

**Affiliations:** 1 Department of Obstetrics and Gynecology, Shengjing Hospital of China Medical University, Shenyang, Liaoning, China; 2 Department of Breast Surgery, Panjin Central Hospital, Panjin, Liaoning, China; 3 Neurosurgery Center, People’s Hospital of Liaoning Province, Shenyang, Liaoning, China; 4 Department of Hepatopancreatobiliary Surgery, Cancer Hospital of Dalian University of Technology, Cancer Hospital of China Medical University, Liaoning Cancer Hospital & Institute, Shenyang, Liaoning, China; 5 Department of Pathology, College of Basic Medical Science, China Medical University, Shenyang, Liaoning, China; 6 Markey Cancer Center, University of Kentucky, Lexington, KY, United States; 7 Department of Colorectal Surgery, Shengjing Hospital of China Medical University, Shenyang, Liaoning, China

**Keywords:** Ki67 proliferation index, breast cancer, neoadjuvant chemotherapy, prognosis, nomogram, cutoff value

## Abstract

**Background:**

The rising incidence of breast cancer underscores the need for precise prognostic assessment following neoadjuvant chemotherapy (NAC). Ki67 is widely utilized for prognostic evaluation. However, its clinical applicability remains debated, particularly regarding the optimal cutoff threshold. This study aims to establish the optimal Ki67 cutoff value and evaluate its prognostic significance in predicting survival outcomes in patients with breast cancer undergoing NAC.

**Methods:**

A retrospective analysis was performed on 255 patients with breast cancer who received NAC between 2011 and 2024. The optimal Ki67 cutoff value was determined using maximally selected rank statistics. Kaplan–Meier survival analysis was used to evaluate the impact of Ki67 on disease-free survival (DFS) and overall survival (OS). Prognostic variables were selected via Cox regression analysis combined with LASSO dimensionality reduction. Based on these findings, nomogram models incorporating Ki67 and other clinical parameters were constructed to predict 1-year, 3-year, and 5-year DFS and OS, and the models were subsequently evaluated.

**Results:**

As a continuous variable, Ki67 presented an increasing and non-linear association with the risk of DFS. Using 20% as the threshold, survival analysis indicated that patients with a high Ki67 proliferation index (Ki67 > 20%) had significantly shortened DFS and OS compared to those with low Ki67 proliferation index. Cox regression analysis also confirmed that Ki67 was a common independent prognostic predictor for both DFS and OS. The nomogram model integrating Ki67, T stage, N stage, and other clinical parameters exhibited strong predictive performance, with the area under the curve (AUC) exceeding 0.900 at all-time points. Calibration plot further validated the model's accuracy, with a C-index of 0.894 for DFS and 0.788 for OS.

**Conclusions:**

A Ki67 cutoff of 20% serves as a reliable predictor of DFS and OS in patients with breast cancer receiving NAC. The developed nomogram models, incorporating Ki67 and other clinical parameters, provide an accurate and clinically valuable tool for individualized prognostic assessment.

## 1 Introduction

In recent years, the global incidence of breast cancer has continued to rise, surpassing lung cancer as the leading malignancy and a major health threat to women (1–3). As a highly heterogeneous disease, breast cancer exhibits diverse risk factors, variable clinical behaviors, distinct molecular profiles, and differential responses to treatment (4, 5). Advances in medical science have expanded therapeutic strategies, encompassing surgery, radiotherapy, chemotherapy, hormone therapy, targeted therapy, and immunotherapy. Among these, neoadjuvant chemotherapy (NAC), a systemic treatment administered preoperatively, has been widely utilized for locally advanced and high-risk early-stage breast cancer (6–8). The primary objectives of NAC include tumor downstaging, increasing the feasibility of breast-conserving surgery, and evaluating therapeutic responses to inform subsequent individualized treatment strategies (9, 10). Despite its critical roles in breast cancer management, accurately predicting prognosis following NAC remains a significant clinical challenge, underscoring the need for reliable prognostic biomarkers to optimize treatment decisions.

Ki67 is a nuclear protein closely associated with cell proliferation, expressed in actively dividing cell nuclei but absent in quiescent (G0 phase) cells (11, 12). In breast cancer, the Ki67 index serves as a key proliferative marker, reflecting tumor cell proliferation rates and contributing to molecular subtyping (13). It has been extensively investigated for its prognostic significance and is regarded as a critical biomarker for assessing tumor biology. High Ki67 proliferation index has been consistently linked to poor survival outcomes in breast cancer. For example, Inwald et al. demonstrated a negative correlation between the Ki67 proliferation index and both disease-free survival (DFS) and overall survival (OS) (14). Similarly, other studies have reported an inverse relationship between Ki67 proliferation index and cancer-specific survival (CSS) in early-stage luminal breast cancer (15). Furthermore, a meta-analysis indicated that among patients receiving neoadjuvant endocrine therapy, high Ki-67 levels were associated with poorer survival outcomes (16). Beyond its prognostic value, Ki67 is also utilized to evaluate the efficacy of NAC, as elevated Ki67 proliferation index generally correlates with poorer treatment responses (17). Moreover, changes in Ki67 levels before and after NAC could serve as dynamic biomarkers for assessing treatment response (18, 19). A latest clinical study has found that neoadjuvant chemotherapy has a better response when the Ki67 index is ≥19% through the analysis of 191 cases of invasive breast cancer (20). Despite substantial evidence supporting Ki67's prognostic utility, its clinical application remains controversial, primarily due to the variability in cutoff values across studies. Different thresholds have been proposed, reflecting tumor heterogeneity and subtype-specific differences. For instance, in patients with breast cancer who failed to achieve pathological complete response (PCR) following neoadjuvant systemic therapy (NST), a study using a 30% cutoff demonstrated that both Ki67C (percentage change in Ki67 before and after NST) and Ki67T (post-surgical Ki67 index) were independent predictors of DFS (21). Another study in Luminal B [human epidermal growth factor receptor 2 (HER2)-negative] breast cancer identified a residual tumor Ki67 threshold of 23%, above which DFS outcomes were significantly worse (22). Additionally, a meta-analysis of 7,716 patients with triple-negative breast cancer (TNBC) found that Ki67 levels exceeding 40% were associated with increased recurrence and mortality risk (23). These findings raise a critical question: Is there a universal Ki67 cutoff applicable to all patients with breast cancer, or should distinct thresholds be established for different molecular subtypes? Optimizing the clinical utility of Ki67 and defining its optimal cutoff value are essential for enhancing the accuracy of breast cancer prognostic assessment.

Numerous studies have explored the integration of Ki67 with other clinicopathological parameters to construct multifactorial prognostic models, thereby improving the accuracy of survival predictions (24–27). For instance, Yu et al. developed a nomogram incorporating Ki67 and additional biomarkers to assess recurrence risk in patients with luminal breast cancer over 50 years old, addressing the limitations faced by that ineligible for 21-gene testing (28). These findings suggest that Ki67 alone may be insufficient for comprehensive prognostic evaluation, and its combination with other critical clinical indicators could enhance predictive precision. Unlike previous studies that relied mainly on fixed or empirical Ki67 thresholds (such as 14%, 20%, or 30%) to stratify prognosis, our study adopts a data-driven approach using maximally selected rank statistics with bootstrap validation to identify a stable cutoff point for both DFS and OS. Furthermore, the combined prognostic value of Ki67 and multiple clinical parameters is assessed, facilitating the development of novel nomograms to improve the predictive accuracy of 1-, 3-, and 5-year DFS and OS. These methodological refinements aim to establish a precise prognostic tool to support individualized treatment strategies and optimize clinical decision-making for NAC-treated patients with breast cancer.

## 2 Methods

### 2.1 Basic characteristics of patients with breast cancer who received NAC

This retrospective study included patients with breast cancer diagnosed and treated at China Medical University Affiliated Hospital between January 2011 and May 2024. The inclusion criteria were as follows: (1) female patients aged 18 to 75 years; (2) histopathologically confirmed invasive ductal carcinoma at initial diagnosis; (3) availability of comprehensive pathological reports, including but not limited to the Ki67 proliferation index, estrogen receptor (ER) status, progesterone receptor (PR) status, and HER2 status; (4) receipt of standard NAC with complete treatment records, administered according to the National Comprehensive Cancer Network (NCCN) guidelines relevant to the year of diagnosis; (5) completion of appropriate surgical treatment based on clinical assessment; and (6) a minimum of one year of follow-up data. NAC regimens included EC-T (epirubicin and cyclophosphamide for four cycles, followed by docetaxel for four cycles), TEC (docetaxel, epirubicin, and cyclophosphamide for six cycles), TP (docetaxel and cisplatin for six cycles), and TC (docetaxel and cyclophosphamide for six cycles). Additionally, HER2-positive patients received trastuzumab with or without pertuzumab as part of their treatment. Exclusion criteria were as follows: (1) history of other malignancies or metastatic tumors; (2) prior chemotherapy or radiotherapy before study initiation; and (3) incomplete clinical records or follow-up data. The study was approved by the hospital's ethics committee.

### 2.2 Data collection

Clinical and pathological data were extracted from electronic medical records, encompassing age, the Ki67 proliferation index, tumor size (T stage), lymph node status (N stage), histologic grade, molecular subtype, and treatment factors (radiotherapy/chemotherapy/neoadjuvant response, etc.). Data integrity and accuracy were independently verified by two reviewers. Patients underwent routine follow-up every three months post-surgery through outpatient visits or telephone interviews to document survival status and recurrence. DFS was defined as the interval from surgery to recurrence or distant metastasis, while OS was measured from surgery to death. For patients without recorded events, follow-up duration was documented as the date of the last contact. Missing data were handled using a complete-case approach. Patients with incomplete baseline, treatment, or outcome data were not eligible for inclusion in the final dataset.

### 2.3 Immunohistochemistry (IHC) staining and Ki67 proliferation index assessment

Postoperatively resected breast cancer tissue samples were fixed in formalin, embedded in paraffin, and sectioned at a thickness of 4 μm. Immunohistochemical staining was conducted using the streptavidin-biotin-peroxidase (SP) method. The sections were de-paraffinized in xylene, rehydrated through a graded ethanol series, and subjected to antigen retrieval. Incubation with a primary antibody against Ki67 (Roche, Confirm Anti-Ki67 Rabbit Monoclonal Primary Antibody) was performed at 4°C overnight, followed by treatment with a secondary antibody, streptavidin-HRP, and diaminobenzidine (DAB) chromogen to visualize antigen-antibody reactions. Hematoxylin was used for nuclear counterstaining. Ki67 proliferation index was independently assessed by two pathologists blinded to clinical outcomes, with quantification based on the percentage of Ki67-positive tumor cells in at least 500 tumor cells across five randomly selected high-power fields (200× magnification).

### 2.4 Statistical analysis

All statistical analyses were performed in R (version 4.4.2). The primary packages included survival, rms, survminer, glmnet, timeROC, pROC, pec, riskRegression, rmda, and ggplot2. The primary study endpoints were DFS and OS. The basic patient characteristics were summarized descriptively: continuous variables as mean ± standard deviation, and categorical variables as counts and percentages. Between-group differences were assessed using t/Wilcoxon tests or Fisher's exact tests. Furthermore, we identified the optimal Ki67 cutoff using maximally selected rank statistics and modeled the potential continuous relationship between Ki67 and recurrence risk using restricted cubic splines (RCS). Univariable and multivariable regression analyses were conducted with Cox proportional hazards models, and results are reported as hazard ratios (HRs) with 95% confidence intervals (CIs). For variable selection prior to nomogram construction, candidates were first screened by univariable Cox regression with an entry criterion of *P* < 0.20. To further mitigate overfitting and enhance model robustness, we also applied LASSO regression for variable preselection. Variables retained by multivariable Cox and LASSO were then integrated, with potential multicollinearity evaluated and excluded as appropriate, to determine the final predictor set for constructing nomograms to predict the 1-, 3-, and 5-year DFS and OS. Model performance was evaluated using the C-index, time-dependent AUC, calibration plots, and decision curve analysis.

## 3 Results

### 3.1 The basic clinicopathological characteristics of the included patients in this study

All the enrolled patients had complete baseline pathological and treatment data, with follow-up for DFS and OS. Distributions of age, T/N stage, histologic grade, molecular subtype, and treatment factors (radiotherapy/chemotherapy/neoadjuvant response, etc.) were summarized in Supplementary Table S1. Over the follow-up period, 55 DFS related events and 26 OS related events were observed.

### 3.2 Identification of the optimal cutoff value of the Ki67 proliferation index for patients with breast cancer receiving NAC

**Figure 1:**
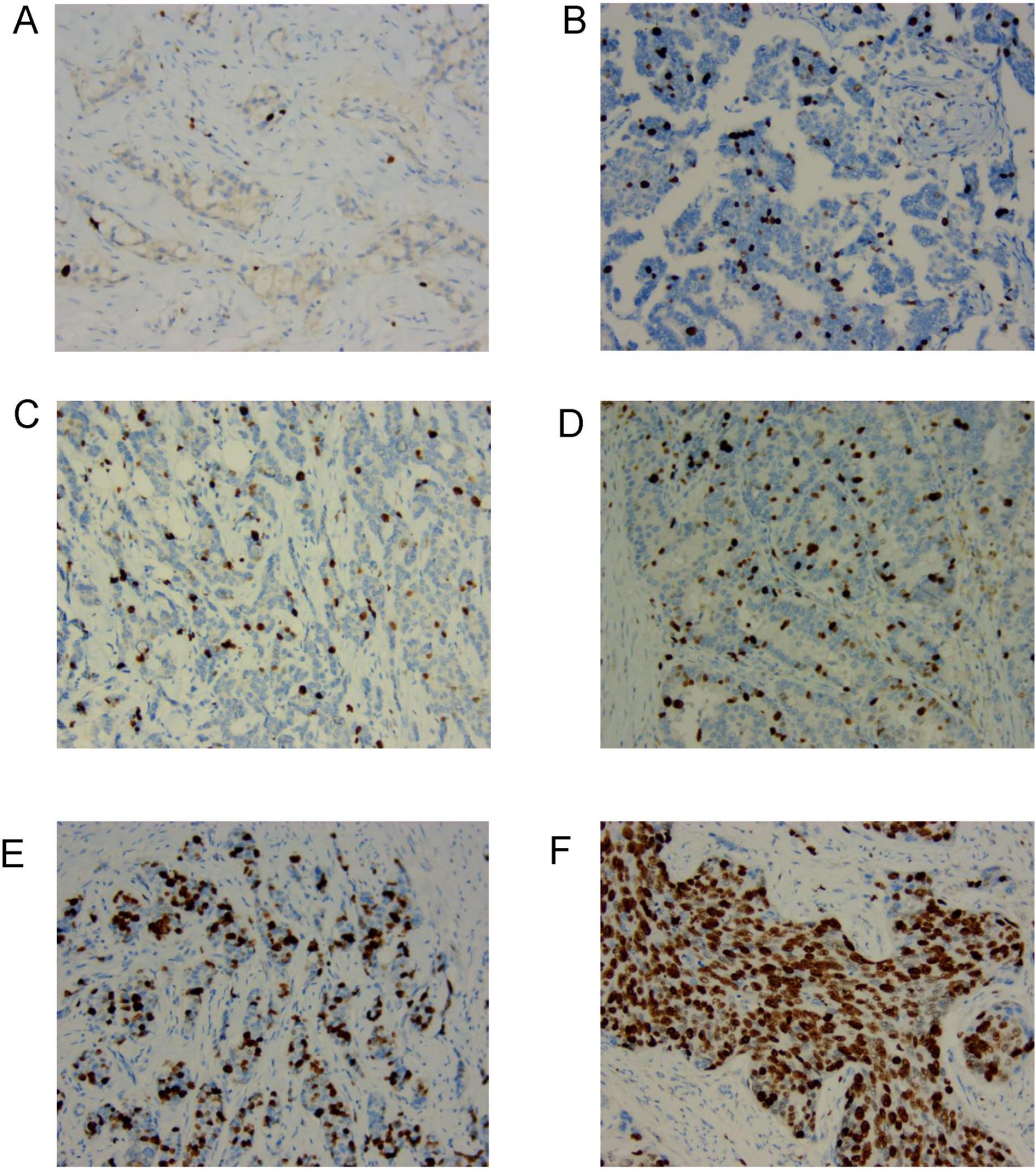
The typical pictures of Ki67 proliferation index in the clinical samples from patients with breast cancer undergoing NAC. **(A–C)** Low Ki67 proliferation index (≤20%) with weak nuclear staining (200×). **(D–F)** High Ki67 proliferation index (>20%) with strong nuclear staining (200×).

Ki67 proliferation index was assessed by IHC in breast cancer samples from patients who underwent NAC. Representative Ki67 staining images are shown in Figure 1. Tumors with low Ki67 proliferation index (≤20%) exhibited sparse nuclear staining (Figures 1A–C), whereas those with high Ki67 proliferation index (>20%) displayed a substantial proportion of positively stained nuclei (Figures 1D–F).

**Figure 2:**
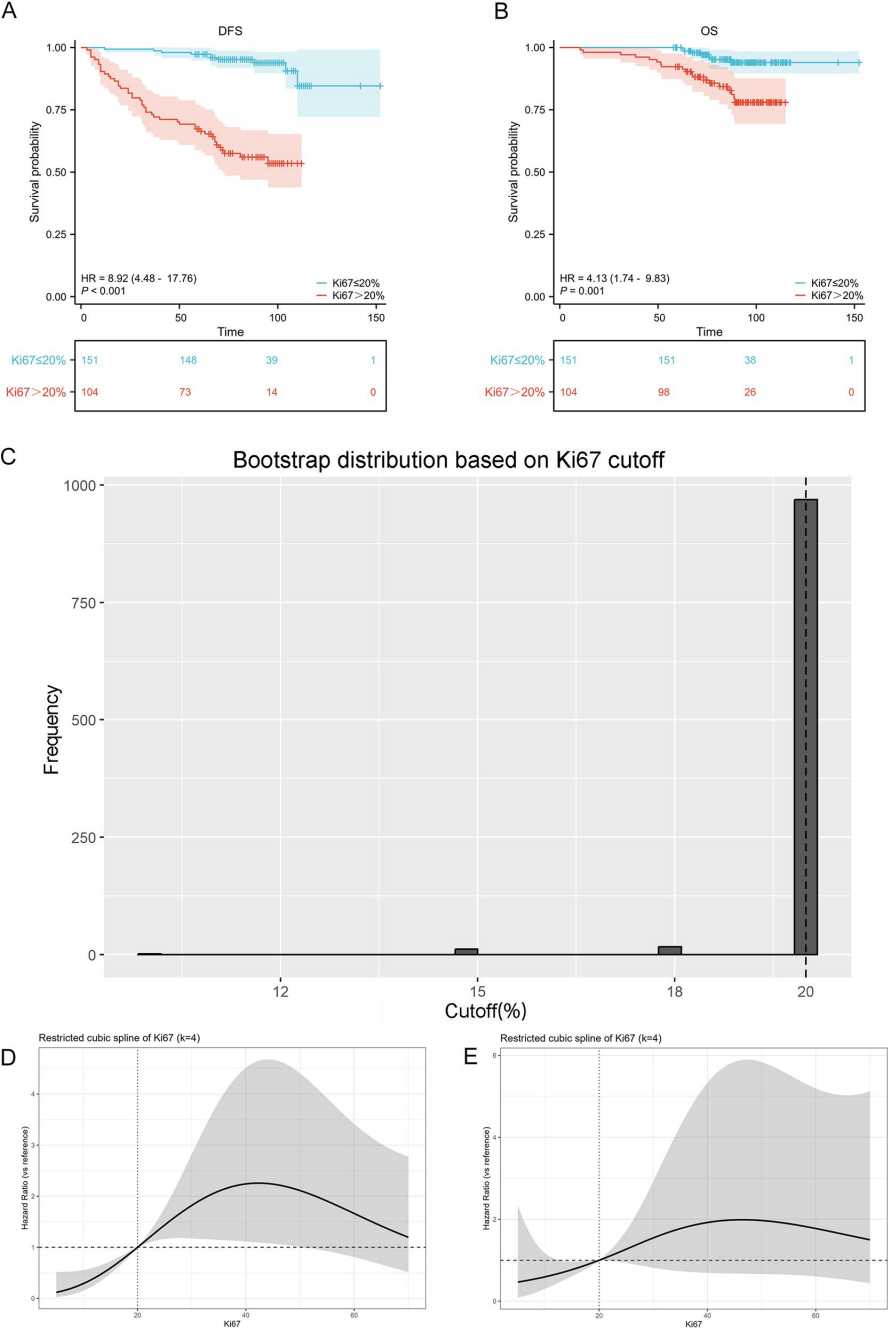
Optimal cutoff determination for the Ki67 index in patients with breast cancer undergoing NAC. **(A)** KM survival curve for DFS. **(B)** KM survival curve for OS. **(C)** Bootstrap distribution based on Ki67 cutoff. **(D)** RCS of Ki67 proliferation index for DFS. **(E)** RCS of Ki67 proliferation index for OS.

Furthermore, using the maximally selected rank statistics, we identified Ki67 = 20% as the optimal cutoff value, which was the most strongly associated with survival differences for DFS and OS. After dichotomizing Ki67 into ≤20% group and >20% group, Kaplan–Meier curves showed clear separation for DFS (*P* < 0.001, HR = −8.92, 95% CI: 4.48–17.76), with the >20% group exhibiting substantially worse DFS; the OS results were directionally consistent but of smaller magnitude (*P* = 0.001, HR = 4.13, 95% CI: 1.74–9.83) (Figures 2A,B). To reduce optimism from selecting and evaluating the cutoff value within the same dataset, we performed B = 1,000 bootstrap resamples for the maxstat-derived cutoff. The resulting distribution was tightly centered at 20%, with only a few resamples near 15% and 18%, indicating the high stability for the 20% threshold (Figure 2C).

### 3.3 Nonlinear relationship of Ki67 as a continuous predictor for the survival outcomes of patients receiving NAC

When Ki67 was entered as a continuous covariate in multivariable Cox models and fitted using restricted cubic splines (RCS, k = 4), the risk curves for both DFS and OS showed a similar pattern: an inflection around 20%, where the curve intersected the HR = 1 line, a progressive increase in risk between ∼20% and 40%–50%, reaching a peak around 40%–50% (more pronounced for DFS than OS), and a subsequent plateau beyond ∼50%–60%. The confidence bands widened markedly at higher Ki67 values, suggesting fewer observations at the extremes and increased estimation uncertainty (Figures 2D,E).

### 3.4 Basic clinicopathological characteristics of the included patients with breast cancer receiving NAC

The 255 included patients were stratified into the group of low Ki67 proliferation index (*n* = 151; Ki67 ≤ 20%) and the group of high Ki67 proliferation index (*n* = 104; Ki67 > 20%) groups (Table 1). The mean ages of the two groups were 50.5 ± 8.8 years and 49.3 ± 9.1 years, respectively, with no statistically significant difference (*P* = 0.301). Histological grading differed significantly between the two groups (*P* = 0.037), with 88.1% of patients in the low Ki67 proliferation index group classified as grade II, compared to 92.3% in the high Ki67 proliferation index group. Molecular subtype analysis revealed a highly significant difference (*P* < 0.001); 79.4% of patients in the low Ki67 proliferation index group were classified as luminal subtype, whereas only 58.6% of those in the high Ki67 proliferation index group fell into this category. In survival analysis, the median DFS was significantly longer in the low Ki67 proliferation index group [87.1 months, interquartile range (IQR): 74.4–99.8] than in the high Ki67 proliferation index group (71.0 months, IQR: 33.3–90.2, *P* < 0.001). However, median OS did not differ significantly between the two groups, with values of 87.2 months (IQR: 74.6–100.2) and 87.4 months (IQR: 70.9–99.3) for the low and high Ki67 proliferation index groups, respectively (*P* = 0.278).

**Table 1:** Baseline characteristics of the included patients based on the level of Ki67 proliferation index.

Characteristics	Ki67 ≤ 20%	Ki67 > 20%	*P*-value
N	151	104	
Age, mean ± sd	50.5 ± 8.8	49.3 ± 9.1	0.301
Menopause, *n* (%)			0.917
Yes	60 (39.7%)	42 (40.4%)	
No	91 (60.3%)	62 (59.6%)	
T_stage, *n* (%)			0.952
T1	9 (6%)	7 (6.7%)	
T2	117 (77.5%)	79 (76%)	
T3	25 (16.6%)	18 (17.3%)	
N_stage, *n* (%)			0.104
N0	61 (40.4%)	38 (36.5%)	
N1	63 (41.7%)	39 (37.5%)	
N2	17 (11.3%)	10 (9.6%)	
N3	10 (6.6%)	17 (16.3%)	
Histological grade, *n* (%)			0.037
I	9 (6%)	0 (0%)	
II	133 (88.1%)	96 (92.3%)	
III	9 (6%)	8 (7.7%)	
Subtype, *n* (%)			0.001
HR+/HER2-	120 (79.4%)	61 (58.6%)	
TNBC	14 (9.3%)	21 (20.2%)	
HER2+	17 (11.3%)	22 (21.2%)	
Radiotherapy, *n* (%)			0.64
No	80 (53%)	52 (50%)	
Yes	71 (47%)	52 (50%)	
Response, *n* (%)			0.117
Non-pCR	144 (95.4%)	94 (90.4%)	
pCR	7 (4.6%)	10 (9.6%)	
DFS_time, median (IQR)	87.1 (74.4, 99.8)	70.95 (33.4, 90.2)	<0.001
DFS_event, *n* (%)			<0.001
1	10 (6.6%)	45 (43.3%)	
0	141 (93.4%)	59 (56.7%)	
OS_time, median (IQR)	87.2 (74.6, 100.2)	87.35 (70.9, 99.3)	0.278
OS_event, *n* (%)			<0.001
1	7 (4.6%)	19 (18.3%)	
0	144 (95.4%)	85 (81.7%)	
Radiotherapy, *n* (%)			0.64
1	71 (47%)	52 (50%)	
0	80 (53%)	52 (50%)	

### 3.5 Univariate and multivariate Cox regression analysis to identify independent prognostic predictors for the patients with breast cancer who received NAC

Univariate and multivariate Cox regression analysis was also conducted (Tables 2, 3). In the univariate Cox regression analysis, a high Ki67 index was significantly associated with poorer DFS (HR = 8.878, 95% CI: 4.459–17.674, *P* < 0.001). Multivariate analysis further confirmed its role as an independent prognostic factor, with the hazard ratio increasing to 18.555 (95% CI: 8.455–40.724, *P* < 0.001). Similarly, Cox regression analysis for OS indicated that a high Ki67 index was predictive of adverse outcomes in the univariate analysis (HR = 4.130, 95% CI: 1.736–9.827, *P* = 0.001) and remained an independent prognostic factor in the multivariate analysis (HR = 4.061, 95% CI: 1.568–10.515, *P* = 0.004). Lymph node status also exhibited a significant prognostic impact. In the univariate analysis, compared to N0, the HRs for DFS in N1 (*n* = 102), N2 (*n* = 27), and N3 (*n* = 27) were 4.037 (*P* = 0.002), 6.742 (*P* < 0.001), and 18.033 (*P* < 0.001), respectively. In the multivariate analysis, these HRs increased to 3.353 (*P* = 0.012), 6.012 (*P* = 0.002), and 17.222 (*P* < 0.001), respectively. For OS, the univariate analysis yielded HRs of 2.148 (*P* = 0.163), 0.736 (*P* = 0.779), and 8.082 (*P* < 0.001) for N1, N2, and N3, respectively, while the multivariate analysis resulted in HRs of 1.982 (*P* = 0.229), 0.573 (*P* = 0.620), and 4.637 (*P* = 0.009), respectively. These findings highlight that N3 stage is a significant prognostic factor in both univariate and multivariate analyses, with lymph node involvement exerting a substantial impact on DFS but a relatively smaller effect on OS. Regarding tumor size, the univariate analysis indicated that, relative to T1 (*n* = 16), the HR for DFS in T3 (*n* = 43) was 11.411 (*P* = 0.017), which increased to 14.735 (*P* = 0.009) in the multivariate analysis. In contrast, for OS, the univariate analysis yielded an HR of 1.575 (*P* = 0.558), while the multivariate analysis produced an HR of 1.048 (*P* = 0.955), indicating that T stage significantly influences DFS but has a limited effect on OS.

**Table 2:** Univariate and multivariate cox regression analysis of clinicopathological risk factors for DFS Among these patients.

Characteristics	Total (N)	Univariate analysis		Multivariate analysis	
		Hazard ratio (95% CI)	*P* value	Hazard ratio (95% CI)	*P* value
Age	255				
<50	115	Reference			
50–59	104	0.942 (0.528–1.680)	0.839		
≥60	36	1.213 (0.564–2.613)	0.621		
Menopause	255				
0	153	Reference			
1	102	1.242 (0.730–2.114)	0.424		
T_stage	255				
T1	16	Reference		Reference	
T2	196	2.263 (0.308–16.615)	0.422	1.984 (0.264–14.891)	0.505
T3	43	11.411 (1.543–84.377)	0.017	14.735 (1.935–112.201)	0.009
N_stage	255				
N0	99	Reference		Reference	
N1	102	4.037 (1.634–9.973)	0.002	3.353 (1.304–8.622)	0.012
N2	27	6.742 (2.397–18.965)	<0.001	6.012 (1.961–18.429)	0.002
N3	27	18.033 (7.139–45.549)	<0.001	17.222 (6.430–46.126)	<0.001
Histological_grade	255				
1	9	Reference			
2	229	1.951 (0.269–14.123)	0.508		
3	17	2.288 (0.255–20.504)	0.459		
Ki67	255				
≤20%	151	Reference		Reference	
>20%	104	8.878 (4.459–17.674)	<0.001	18.555 (8.455–40.724)	<0.001
Subtype	255				
HR+/HER2-	181	Reference			
HER2+	39	1.294 (0.643–2.604)	0.469		
TNBC	35	1.041 (0.485–2.237)	0.917		
Radiotherapy	255				
0	132	Reference		Reference	
1	123	1.708 (0.995–2.930)	0.052	0.938 (0.527–1.669)	0.828
Response	255				
pCR	17	Reference			
Non-pCR	238	1.205 (0.376–3.863)	0.753		

**Table 3:** Univariate and multivariate Cox regression analysis of clinicopathological risk factors for OS Among these patients.

Characteristics	Total (*N*)	Univariate analysis		Multivariate analysis	
		Hazard ratio (95% CI)	*P* value	Hazard ratio (95% CI)	*P* value
Age	255				
<50	115	Reference			
50–59	104	1.140 (0.484–2.686)	0.764		
≥60	36	1.529 (0.522–4.479)	0.438		
Menopause	255				
0	153	Reference			
1	102	1.209 (0.559–2.616)	0.629		
T_stage	255				
T1	16	Reference		Reference	
T2	196	0.505 (0.115–2.224)	0.366	0.338 (0.070–1.629)	0.176
T3	43	1.575 (0.344–7.203)	0.558	1.048 (0.205–5.343)	0.955
N_stage	255				
N0	99	Reference		Reference	
N1	102	2.148 (0.734–6.286)	0.163	1.982 (0.651–6.036)	0.229
N2	27	0.736 (0.086–6.297)	0.779	0.573 (0.063–5.178)	0.62
N3	27	8.082 (2.761–23.652)	<0.001	4.637 (1.469–14.632)	0.009
Histological_grade	255				
1	9	Reference		Reference	
2	229	0.714 (0.096–5.319)	0.743	0.294 (0.032–2.675)	0.277
3	17	2.306 (0.258–20.642)	0.455	1.028 (0.095–11.091)	0.982
Ki67	255				
≤20%	151	Reference		Reference	
>20%	104	4.130 (1.736–9.827)	0.001	4.061 (1.568–10.515)	0.004
Subtype	255				
HR+/HER2-	181	Reference		Reference	
HER2+	39	2.826 (1.205–6.626)	0.017	2.391 (0.954–5.992)	0.063
TNBC	35	0.619 (0.142–2.693)	0.523	0.546 (0.114–2.612)	0.449
Radiotherapy	255				
0	132	Reference			
1	123	1.160 (0.536–2.509)	0.706		
Response	255				
pCR	17	Reference			
Non-pCR	238	0.955 (0.225–4.043)	0.95		

### 3.6 Construction and validation of the nomograms predicting the survival outcomes of patients with breast cancer who received NAC based on Ki67 and other clinical indicators

**Figure 3:**
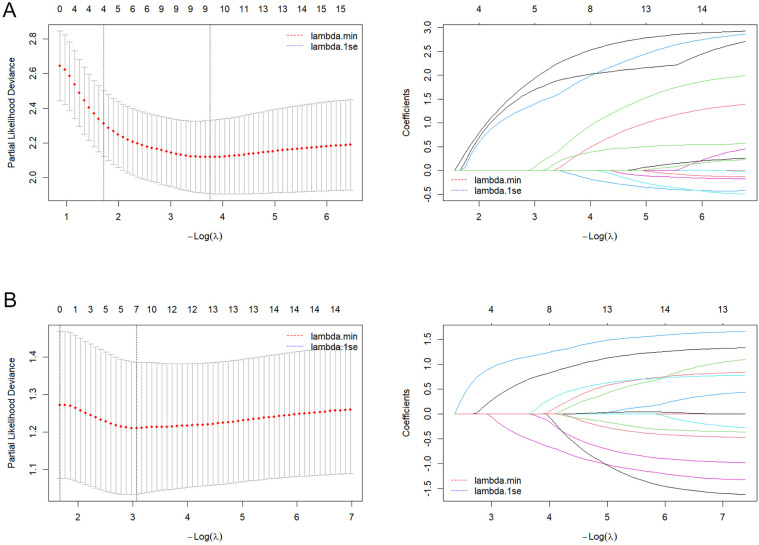
LASSO-based feature preselection and parameter tuning. **(A)** DFS model: left, cross-validation curve; right, LASSO coefficient path plot. **(B)** OS model: left, cross-validation curve; right, LASSO coefficient path plot.

**Figure 4:**
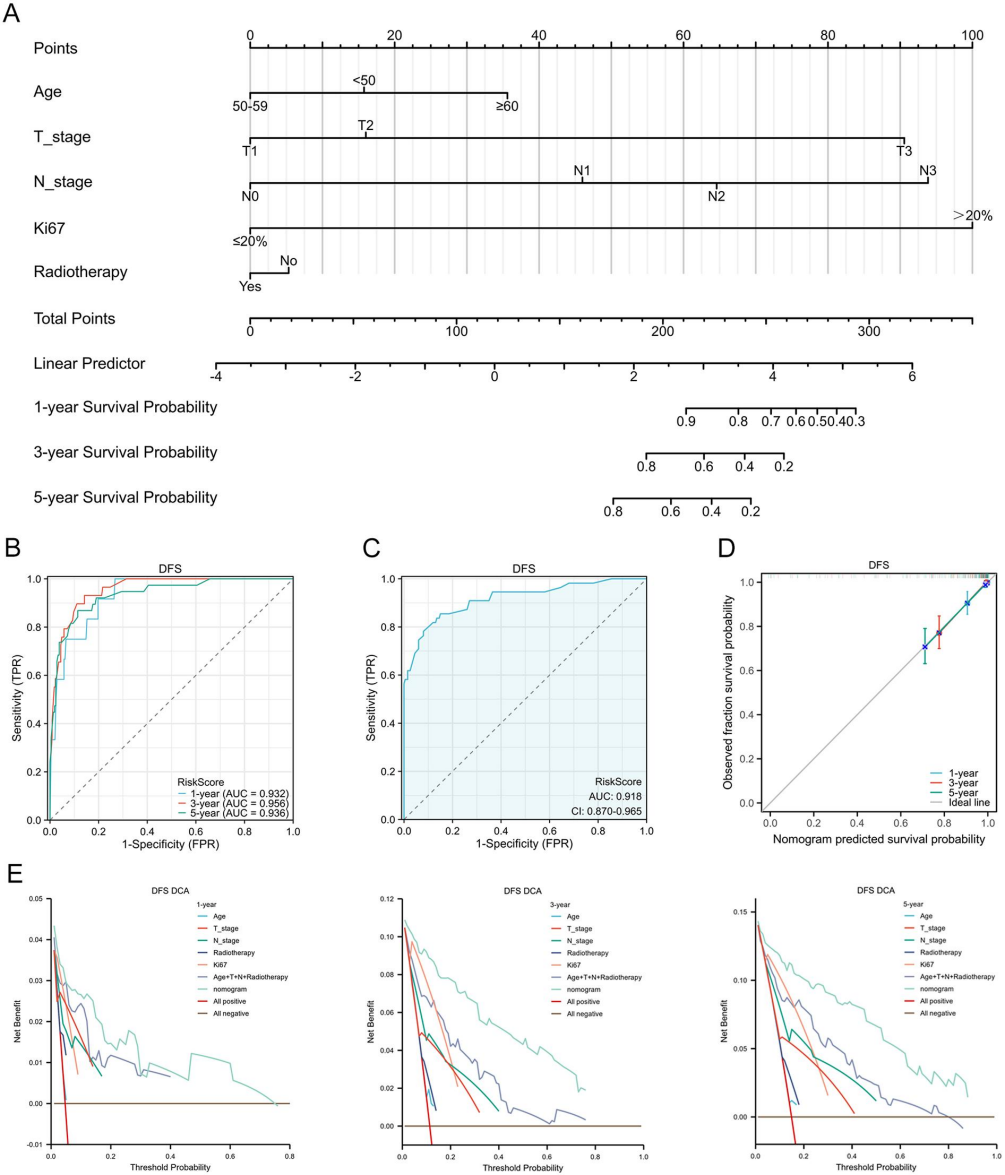
Development and validation of a nomogram for DFS prediction in patients with breast cancer receiving NAC. **(A)** Nomogram integrating multiple prognostic factors to predict DFS. **(B)** timeROC curve assessing the nomogram's predictive performance. **(C)** Diagnostic ROC curve of the nomogram. **(D)** Calibration curves for 1-, 3-, and 5-year DFS. **(E)** Decision curve analysis at 1-, 3-, and 5-year.

Based on candidate predictors identified by univariable screening (*P* < 0.20) for DFS and OS, we further performed dimensionality reduction via LASSO (Figures 3A,B). Incorporating clinical plausibility and collinearity assessment, we further constructed the nomograms to predict 1-, 3-, and 5-year DFS and OS for these patients. The final DFS nomogram included Age, T stage, N stage, Ki67, and Radiotherapy (Figure 4A), whereas the OS nomogram included T stage, N stage, Ki67, Histological grade, and Subtype (Supplementary Figure S1A). Variables consistently retained across repeated cross-validation were concordant with prior clinical evidence, and all final effect sizes were re-estimated within multivariable Cox models (Supplementary Tables S2, S3). After internal validation with B = 1,000 bootstrap resamples, both nomograms demonstrated time-dependent ROC performance at 1, 3, and 5 years that was consistent with the apparent estimates: the AUC values for DFS were 0.932, 0.956, and 0.936 (Figure 4B), and the AUC values for OS were 0.930, 0.924, and 0.898 (Supplementary Figure S1B). The AUC value of diagnostic ROC was 0.918 (95% CI, 0.870–0.965) for DFS and 0.808 (95% CI, 0.700–0.915) for OS (Figure 4C, Supplementary Figure S1C). Corresponding calibration plots indicated overall agreement between predicted probabilities and observed risks, with only mild deviation at the high-risk end (Figure 4D, Supplementary Figure S1D). In decision curve analysis, the models including Ki67 yielded consistently greater net benefit than the baseline model, supporting stable and clinically meaningful utility across typical threshold probabilities (Figure 4E, Supplementary Figure S1E). In addition, proportional hazards assumptions were satisfied for both final models based on Schoenfeld residual tests, with strong discrimination for DFS (C-index = 0.894; 95% CI, 0.872–0.915) and moderate-to-good discrimination for OS (C-index = 0.788; 95% CI, 0.735–0.841) (Supplementary Table S4).

## 4 Discussion

Breast cancer remains one of the most prevalent malignancies affecting women worldwide (29, 30), ranking second in incidence and fifth in mortality among Chinese women (31). Despite its high incidence, the overall mortality risk is relatively low, suggesting that patients undergoing standardized treatment regimens—including NAC, surgery, and adjuvant therapy—can achieve prolonged survival and improved quality of life. The integration of these treatment modalities has significantly improved clinical outcomes. However, the pronounced heterogeneity of breast cancer results in considerable variability in patient responses. This underscores the necessity of identifying reliable prognostic biomarkers and clinical factors that influence survival outcomes. Among potential prognostic indicators, the Ki67 index has garnered attention as a key marker of tumor cell proliferation, making it a strong candidate for assessing survival and recurrence patterns in NAC-treated patients with breast cancer.

In this retrospective study, for breast cancer patients who received NAC, we systematically evaluated the continuous risk gradient of Ki67, the clinical feasibility of the 20% threshold, and its predictive value for DFS and OS. We obtained the following results: After adjusting for confounding factors such as T/N stage and age, Ki67 (as a continuous variable) showed a monotonically increasing and non-linear association with DFS. Using 20% as an empirical threshold for binary classification also demonstrated interpretability and feasibility. In terms of prognostic significance, patients with a high Ki67 proliferation index (>20%) had significantly poorer DFS and OS compared to those with low Ki67 proliferation index. Finally, through multivariate Cox regression analysis and LASSO dimensionality reduction, the study constructed novel nomogram models incorporating variables including Ki67 index, T stage, and N stage, providing an individualized prognostic assessment tool for breast cancer patients receiving NAC.

Ki67, a key proliferation index biomarker, plays a pivotal role in various malignancies, particularly breast cancer, where it serves multiple functions, including prognostication, prediction of response to chemotherapy and endocrine therapy, and as a dynamic marker for NAC efficacy (32–35). However, the optimal cutoff value for Ki67 varies depending on its specific clinical application. For instance, the St. Gallen Consensus recommends a 14% threshold to differentiate between Luminal A and Luminal B subtypes and to predict endocrine therapy responsiveness (36). In contrast, the International Ki67 in Breast Cancer Working Group (IKBCWG) categorizes tumors with a Ki67 index below 5% as “low” and those with an index of 30% or higher as “high” (37). A study from the Fudan University Shanghai Cancer Center demonstrated that a Ki67 cutoff of 30% provides early independent prognostic value for OS and DFS in TNBC, particularly in stage I patients, where Ki67 > 30% is significantly associated with poorer prognosis (38). By integrating long-term follow-up data, this study initially identified 20% as a robust cutoff applicable across different breast cancer subtypes in patients receiving NAC. This threshold effectively stratified DFS and exhibited strong discriminatory power for OS. A real-world study involving 956 patients with HR+/HER2- breast cancer similarly identified 22.5% as a critical threshold, with patients exhibiting Ki67 ≥ 22.5% facing a higher risk of early recurrence and metastasis (39). However, our study further adjusted the cutoff value to 20% using the maximally selected rank statistics method, which more effectively differentiates clinical risk profiles and aligns with routine pathology reporting, thereby enhancing its practical applicability in prognostic assessment.

The nomogram developed in this study provides clinicians with a precise tool for individualized prognostic assessment in patients with breast cancer receiving NAC. In recent years, the construction and application of nomograms have gained considerable attention in breast cancer prognostic research. Multiple studies have emphasized the importance of integrating diverse biomarkers and clinical imaging data into prognostic models, particularly for optimizing adjuvant treatment strategies in early-stage breast cancer (40–45). One study demonstrated that nomograms enable personalized prognostic evaluation by incorporating multiple variables, with significant applications in patients with triple-negative breast cancer (46). Similarly, this study constructed a survival prediction nomogram based on a multivariate regression model, confirming the independent prognostic significance of key factors such as Ki67 proliferation index, tumor size, and lymph node status. Unlike models that only include independent predictive variables, this nomogram also integrates two variable selection methods to construct separate nomograms for predicting DFS and OS, respectively. The models, which incorporate variables including Ki67 index, T stage, N stage, and other clinical variables, demonstrated high predictive accuracy at the 1-, 3-, and 5-year follow-ups, with nearly all AUCs exceeding 0.90. This finding is consistent with the study by House et al., which demonstrated that Ki67 combined with multiple pathological parameters effectively predicts 3- and 5-year DFS (47), further supporting the clinical utility of this model. Moreover, the calibration curves demonstrated strong predictive concordance, reinforcing the model's reliability. The time-dependent ROC analysis further validated its predictive performance at different time points, with AUC values of 0.932, 0.956, and 0.936 for DFS at 1, 3, and 5 years, respectively, and 0.930, 0.924, and 0.898 for OS at the corresponding time points. These results align with findings from Guan et al., who reported that Ki67, when combined with other clinical variables, enhances long-term prognostic accuracy in a multicenter cohort of patients with breast cancer (48). Comprehensive validation further strengthened the credibility and clinical applicability of the constructed nomogram.

Despite the significant clinical implications of this study, several limitations should be acknowledged. First, this was a single-center retrospective study with a modest sample size and a long accrual period, which may have introduced selection bias and era effects due to evolving diagnostic and treatment standards. Although the events-per-variable ratio met standard recommendations, the limited number of OS events may have reduced the precision and robustness of the survival estimates. Second, treatment heterogeneity may have introduced residual confounding that could not be fully controlled. Third, although bootstrap internal validation was performed to reduce model overfitting, external validation using independent multicenter datasets remains lacking. To improve generalizability, a multicenter evaluation involving patients from different calendar eras and institutions is underway. Fourth, interlaboratory variability in Ki67 detection and scoring may affect reproducibility and limit its universal applicability. Standardized immunohistochemical protocols are therefore essential. Finally, as all patients received neoadjuvant chemotherapy, the findings may not be directly applicable to non-NAC populations. Future prospective multicenter studies with standardized Ki67 assessment and harmonized follow-up protocols are warranted to confirm the robustness and clinical utility of the proposed model.

## 5 Conclusions

This study determined a stable and clinically meaningful Ki67 cutoff value of 20% using maximally selected rank statistics with bootstrap validation in breast cancer patients treated with neoadjuvant chemotherapy. Ki67, whether modeled continuously through restricted cubic splines or dichotomously at 20%, was independently associated with DFS. By integrating Ki67 with traditional clinicopathologic factors, we developed and internally validated a prognostic nomogram that demonstrated good discrimination and calibration in predicting 3- and 5-year DFS. Nevertheless, as this was a retrospective study, external validation in independent cohorts and prospective trials is warranted to confirm the generalizability of the cutoff value and the predictive performance of the nomogram. Future research should also explore automated or standardized Ki67 quantification methods to further enhance reproducibility and clinical applicability.

## Data Availability

The original contributions presented in the study are included in the article/Supplementary Material, further inquiries can be directed to the corresponding author/s.
