# Supplementary material for: Defining the optimal Ki67 cutoff values for survival prediction in neoadjuvant chemotherapy-treated patients with breast cancer
Source: Front Surg. 2025 Nov 24;12:1697963. doi: 10.3389/fsurg.2025.1697963 (PMC12682876; doi:10.3389/fsurg.2025.1697963)
Supplement: Supplementary file 1 [file Datasheet1.docx]

Supplementary Table 1. Baseline Characteristics of the Patients Included.

| Characteristics | overall |
| --- | --- |
| Age, mean ± SD | 50.0 ± 8.9 |
| Menopause, n (%) |  |
| Yes | 102 (40%) |
| No | 153 (60%) |
| T_stage, n (%) |  |
| T2 | 196 (76.9%) |
| T1 | 16 (6.3%) |
| T3 | 43 (16.9%) |
| N_stage, n (%) |  |
| N0 | 99 (38.8%) |
| N1 | 102 (40%) |
| N2 | 27 (10.6%) |
| N3 | 27 (10.6%) |
| Histological grade, n (%) |  |
| I | 9 (3.5%) |
| II | 229 (89.8%) |
| III | 17 (6.7%) |
| Ki67, median (IQR) | 20 (10, 40) |
| subtype, n (%) |  |
| HR+/HER2- | 181 (71%) |
| HER2+ | 39 (15.3%) |
| TNBC | 35 (13.7%) |
| Radiotherapy, n (%) |  |
| No | 132 (51.8%) |
| Yes | 123 (48.2%) |
| Response, n (%) |  |
| Non-pCR | 238 (93.3%) |
| pCR | 17 (6.7%) |
| DFS_time, median (IQR) | 83 (67, 97.2) |
| DFS_event, n (%) |  |
| 0 | 200 (78.4%) |
| 1 | 55 (21.6%) |
| OS_time, median (IQR) | 87.2 (73.15, 100) |
| OS_event, n (%) |  |
| 0 | 229 (89.8%) |
| 1 | 26 (10.2%) |
| radiotherapy, n (%) |  |
| 0 | 132 (51.8%) |
| 1 | 123 (48.2%) |

| Characteristics | HR | confidence interval | | | P value |
| --- | --- | --- | --- | --- | --- |
| Age |  |  |  |  |  |
| <50 | Reference |  |  |  |  |
| 50-59 | 0.627 | 0.338 -1.161 | | | 0.138 |
| ≥60 | 1.801 | 0.770-4.210 | | | 0.175 |
| T_stage |  |  |  |  |  |
| T1 | Reference |  |  |  |  |
| T2 | 1.607 | 0.211-12.229 | | | 0.647 |
| T3 | 14.643 | 1.921-111.637 | | | 0.010 |
| N_stage |  |  |  |  |  |
| N0 | Reference |  |  |  |  |
| N1 | 3.908 | 1.489-10.258 | | | 0.006 |
| N2 | 6.781 | 2.179-21.101 | | | 0.001 |
| N3 | 16.140 | 5.914-44.045 | | | **<0.001** |
| Ki67 |  |  |  |  |  |
| ＞20% | Reference |  |  |  |  |
| ≤20% | 0.052 | 0.023-0.114 | | | **<0.001** |
| Radiotherapy |  |  |  |  |  |
| Yes | Reference |  |  |  |  |
| No | 1.171 | 0.649-2.111 | | | 0.600 |

Supplementary Table 2. Multivariate Cox Regression Analysis for the DFS Nomogram.

| Characteristics | HR | confidence interval | P value |
| --- | --- | --- | --- |
| T_stage |  |  |  |
| T1 | Reference |  |  |
| T2 | 0.338 | 0.070-1.629 | 0.176 |
| T3 | 1.048 | 0.205-5.343 | 0.955 |
| N_stage |  |  |  |
| N0 | Reference |  |  |
| N1 | 1.982 | 0.651-6.036 | 0.229 |
| N2 | 0.573 | 0.063-5.178 | 0.620 |
| N3 | 4.637 | 1.469-14.632 | 0.009 |
| histological_grade |  |  |  |
| 1 | Reference |  |  |
| 2 | 0.294 | 0.032-2.675 | 0.277 |
| 3 | 1.028 | 0.095-11.091 | 0.982 |
| Ki67 |  |  |  |
| ≤20% | Reference |  |  |
| ＞20% | 4.061 | 1.568-10.515 | 0.004 |
| subtype |  |  |  |
| HR+/HER2- | Reference |  |  |
| HER2+ | 2.391 | 0.954-5.992 | 0.063 |
| TNBC | 0.546 | 0.114-2.612 | 0.449 |

Supplementary Table 3. Multivariate Cox Regression Analysis for the OS Nomogram.

Supplementary Table 4. Global Statistical Test of the Nomogram.

| Global Statistical | DFS nomogram | | OS nomogram | |
| --- | --- | --- | --- | --- |
|  | Results | P value |  | P value |
| Concordance (C-index (95% CI)) | 0.894(0.872-0.915) |  | 0.788(0.735-0.841) |  |
| Likelihood ratio test | 153.76 | p=<2e-16 | 40.52 | p=1.37e-05 |
| Wald test | 115.96 | p=<2e-16 | 37.86 | p=4.01e-05 |
| Score test(logrank) | 178.86 | p=<2e-16 | 50.35 | p=2.3e-07 |

Supplementary Figure 1


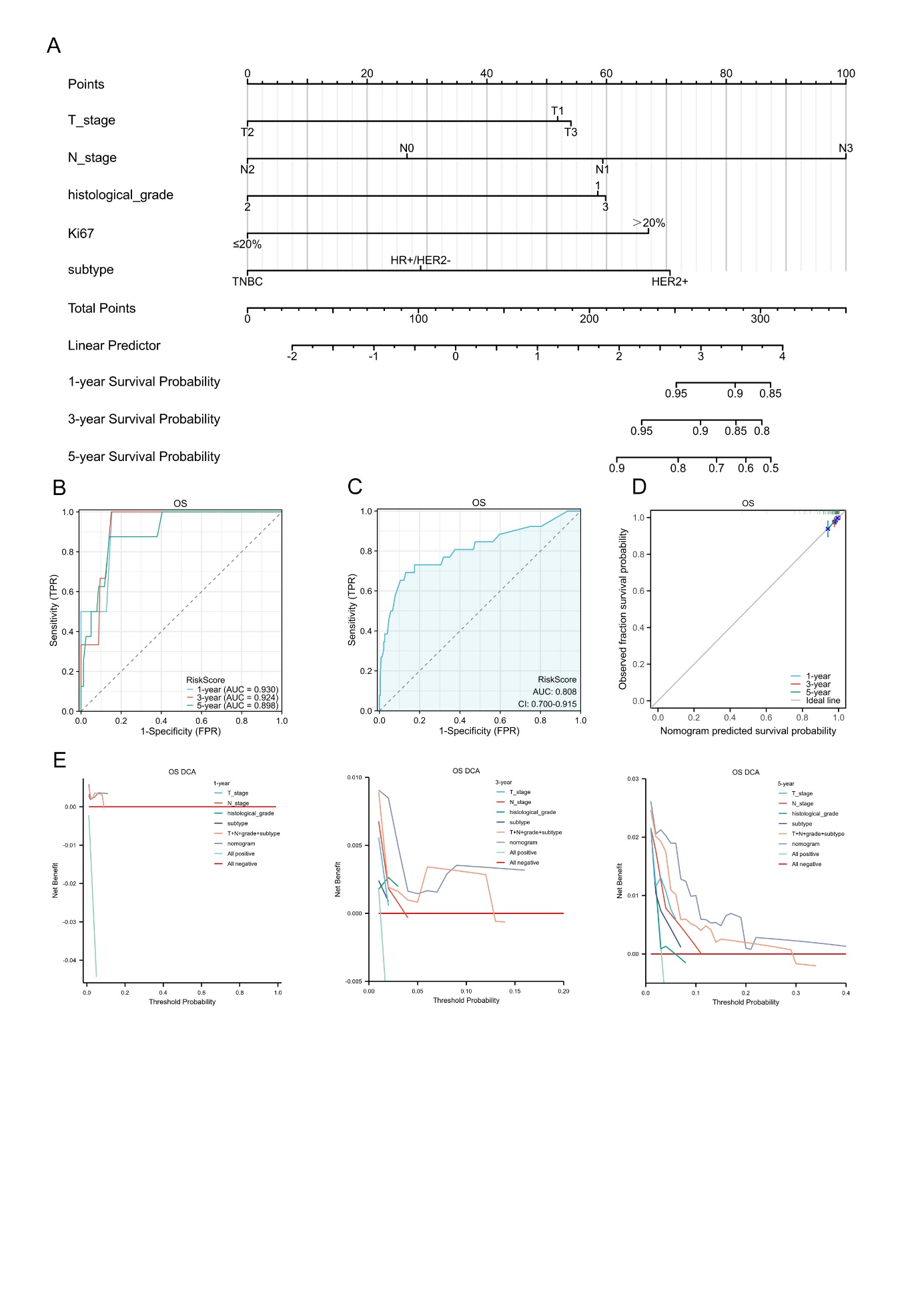


**Supplementary Figure 1. Development and validation of a nomogram for OS prediction in patients with breast cancer receiving NAC.**

(A): Nomogram integrating multiple prognostic factors to predict OS.

(B): timeROC curve assessing the nomogram's predictive performance.

(C): Diagnostic ROC curve of the nomogram.

(D): Calibration curves for 1-, 3-, and 5-year OS.

(E): Decision curve analysis at 1-, 3-, and 5-year.
